# quickARSC: standalone package and web interface for profiling elemental stoichiometry of proteomes

**DOI:** 10.1128/mra.00031-26

**Published:** 2026-03-30

**Authors:** Satoshi Nishino, Kento Tominaga, Yuki Nishimura, Susumu Yoshizawa

**Affiliations:** 1 Department of Integrated Biosciences, Graduate School of Frontier Sciences, The University of Tokyo 34811 , Kashiwa, Chiba, Japan; 2 Atmosphere and Ocean Research Institute, The University of Tokyo 34811 , Kashiwa, Chiba, Japan; 3 Department of Natural Environmental Studies, Graduate School of Frontier Sciences, The University of Tokyo 34811 , Kashiwa, Chiba, Japan; University of Michigan, Ann Arbor, Michigan, USA

**Keywords:** ARSC, computational biology, python package, elemental stoichiometry, web interface

## Abstract

Here, we present quickARSC, a command-line tool computing elemental composition per amino acid residue side chain (ARSC) from amino acid sequences. We also provide the web interface to enable users to access, filter, visualize, and download the pre-computed N-ARSC, C-ARSC, and S-ARSC of 143,614 prokaryotic species.

## ANNOUNCEMENT

Microbial genomes and proteomes are shaped not only by functional requirements but also constrained by the elemental resource landscape (1, 2). These elemental constraints, commonly quantified using genomic guanine-cytosine proportion (GC content) and proteomic elemental composition per amino acid residue side chain (ARSC), serve as proxies for underlying elemental stoichiometry. For example, environmental resource availability drives shifts in genomic base composition to optimize nitrogen (N) investment (3, 4). Similarly, nutrient availability drives shifts in the N-ARSC and C-ARSC of proteomes (2, 5, 6). These stoichiometric traits enable a systematic quantification of nutrient investment across diverse microorganisms.

While GC content can be readily computed using many existing bioinformatics tools, such as SeqKit (7), the routine calculation of ARSC relies on custom in-house scripts (2, 5). The lack of a standardized, high-throughput tool limits the accessibility of stoichiometric profiling, creating a technical barrier to the broader adoption of these analyses into comparative (meta)genomics. To address this, we developed quickARSC, a standalone and platform-independent package that computes N-ARSC, C-ARSC, and S-ARSC from amino acid or nucleotide sequences. Calculations follow established definitions (1, 2, 5), computing average elemental counts per side chain from protein FASTA files (.faa and .faa.gz) or via integrated open reading frame prediction using Prodigal (8) for nucleotide files (e.g., .fna and .fna.gz). quickARSC is designed for high-throughput workflows, employing a multiprocessing module to distribute computations across CPU cores. With minimal core dependencies (only Biopython [9]) and a simple command-line interface, our tool can be readily incorporated into existing bioinformatics pipelines.

**Fig 1:**
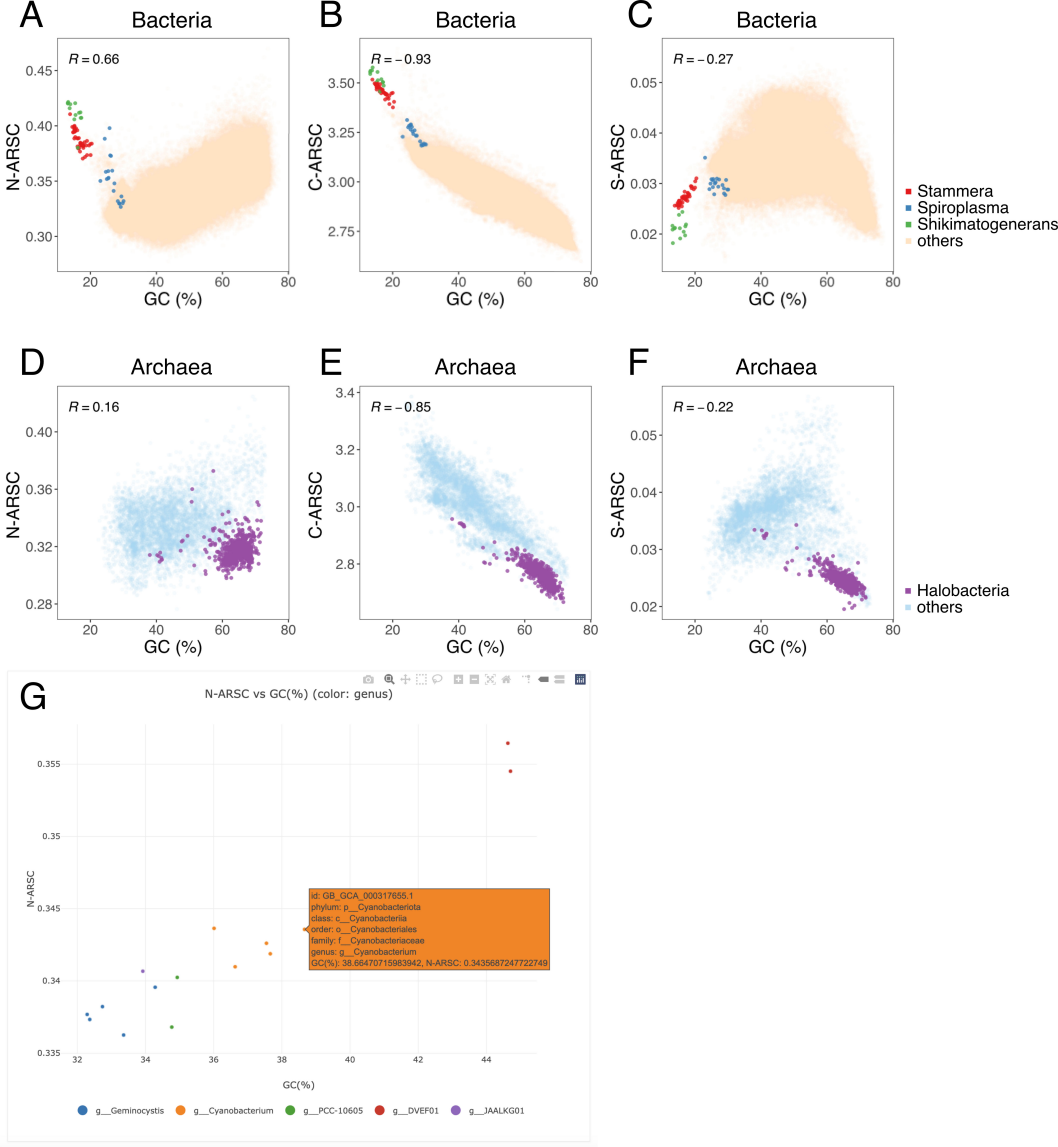
Relationship between genomic GC content and proteomic elemental stoichiometry across 143,614 prokaryotic species and an overview of the quickARSC web interface. Scatter plots display the average number of atoms per amino acid side chain (ARSC) against genomic GC content (%) for 136,646 bacteria and 6,968 archaea. (**A**) Bacterial N-ARSC, (**B**) bacterial C-ARSC, (**C**) bacterial S-ARSC, (**D**) archaeal N-ARSC, (**E**) archaeal C-ARSC, and (**F**) archaeal S-ARSC. Point colors are based on taxonomic information from the Genome Taxonomy Database release r226.0: the genera *Stammera* (red), *Spiroplasma* (blue), and *Shikimatogenerans* (green), and the class *Halobacteria* (purple). Other bacteria (light orange) and archaea (light blue) are shown with semi-transparency. Each panel shows Pearson’s *r*; all correlations are statistically significant (*P* < 0.001, calculated using the R package ggpubr). (**G**) The quickARSC web interface visualizing N-ARSC of the family *Cyanobacteriaceae*.

To demonstrate the utility of quickARSC, we computed ARSC for 143,614 representative prokaryotes from the Genome Taxonomy Database (GTDB) r226.0 (10) (Fig. 1A through F). Consistent with previous studies (2, 11), bacteria exhibited a positive correlation between genomic GC content and N-ARSC (Fig. 1A, Pearson’s *r* = 0.66), and a negative correlation with C-ARSC (Fig. 1B, Pearson’s *r* = −0.93). Bacteria with GC content below 30% (e.g., the genera *Shikimatogenerans*, *Stammera*, and *Spiroplasma*) exhibited elevated N-ARSC as previously reported (2). In contrast, archaea showed no overall correlation between GC content and N-ARSC (Fig. 1D, Pearson’s *r* = 0.16). This decoupling is in line with the acidic proteome of halophilic archaea (12); for instance, the class *Halobacteria* shows high GC contents yet low N-ARSC (Fig. 1D). The S-ARSC peaked within the intermediate GC content range (Fig. 1C and F). This pattern is consistent with expectations based on the standard genetic code (13); specifically, because the codons for Met (ATG) and Cys (TGT and TGC) contain both A/T and G/C bases, their frequencies are influenced by genomic base composition. These results illustrate that quickARSC reliably reproduces previously reported relationships between genomic base composition and proteomic elemental stoichiometry.

The pre-computed N-ARSC, C-ARSC, and S-ARSC are accessible through an interactive web interface (https://stsnsn.github.io/quickARSC/), enabling ARSC analysis of specified lineages (Fig. 1G). Additionally, it performs fast calculations based on a user input amino acid sequence and includes the ability to compare new results with pre-computed data. Our package and web interface allow users to quantify the elemental composition of proteomes and provide insights into nutrient resource allocation.

## Data Availability

quickARSC is openly available under the GNU General Public License (v2.0) on GitHub (https://github.com/stsnsn/quickARSC/) and was tested using Python 3.11.5 and Biopython 1.85. The package is distributed via PyPI (https://pypi.org/project/arsc/). Instructions and usage examples are provided in the GitHub repository. The pre-computed data set for 143,614 representative prokaryotes is available via our GitHub pages (https://stsnsn.github.io/quickARSC/). The visualization R code and the accompanying data set used in this study are archived in Zenodo (https://doi.org/10.5281/zenodo.18758052).

## EDITOR'S NOTE

**Ed. Note**: This article was handled by Pat Schloss, who acted as a guest editor in consultation with the editor in chief, Irene Newton. Dr. Shloss's role as the guest editor of this article is outside of his duties as the chair of the Journals Committee.
